# The molecular profile of synovial fluid changes upon joint distraction and is associated with clinical response in knee osteoarthritis

**DOI:** 10.1016/j.joca.2019.12.005

**Published:** 2020-03

**Authors:** F.E. Watt, B. Hamid, C. Garriga, A. Judge, R. Hrusecka, R.J.H. Custers, M.P. Jansen, F.P. Lafeber, S.C. Mastbergen, T.L. Vincent

**Affiliations:** † Centre for Osteoarthritis Pathogenesis Versus Arthritis, Kennedy Institute of Rheumatology, Roosevelt Drive, Nuffield Department of Orthopaedics, Rheumatology and Musculoskeletal Sciences, University of Oxford, UK; ‡ Centre for Osteoarthritis Pathogenesis Versus Arthritis, Kennedy Institute of Rheumatology, Nuffield Department of Orthopaedics, Rheumatology and Musculoskeletal Sciences, University of Oxford, UK; § Centre for Statistics in Medicine, Nuffield Department of Orthopaedics, Rheumatology and Musculoskeletal Sciences, University of Oxford, UK; ‖ Musculoskeletal Research Unit, University of Bristol, UK; ¶ National Institute for Health Research Bristol Biomedical Research Centre (NIHR Bristol BRC), University Hospitals Bristol NHS Foundation Trust, UK; # MRC Lifecourse Epidemiology Unit, University of Southampton, Southampton General Hospital, Southampton, UK; †† Department of Orthopaedic Surgery, University Medical Center Utrecht, the Netherlands; ‡‡ Department of Rheumatology & Clinical Immunology, University Medical Center Utrecht, the Netherlands

**Keywords:** Osteoarthritis, Orthopaedic, Cytokines, Synovial fluid, Biomarker, Distraction

## Abstract

**Objective:**

Surgical knee joint distraction (KJD) leads to clinical improvement in knee osteoarthritis (OA) and also apparent cartilage regeneration by magnetic resonance imaging. We investigated if alteration of the joint's mechanical environment during the 6 week period of KJD was associated with a molecular response in synovial fluid, and if any change was associated with clinical response.

**Method:**

20 individuals undergoing KJD for symptomatic radiographic knee OA had SF sampled at baseline, midpoint and endpoint of distraction (6 weeks). SF supernatants were measured by immunoassay for 10 predefined mechanosensitive molecules identified in our previous pre-clinical studies. The composite Knee injury and OA Outcome Score-4 (KOOS_4_) was collected at baseline, 3, 6 and 12 months.

**Results:**

13/20 (65%) were male with mean age 54°±°5yrs. All had Kellgren–Lawrence grade ≥2 knee OA. 6/10 analytes showed statistically significant change in SF over the 6 weeks distraction (activin A; TGFβ-1; MCP-1; IL-6; FGF-2; LTBP2), *P* < 0.05. Of these, all but activin A increased. Those achieving the minimum clinically important difference of 10 points for KOOS_4_ over 6 months showed greater increases in FGF-2 and TGFβ-1 than non-responders. An increase in IL-8 during the 6 weeks of KJD was associated with significantly greater improvement in KOOS_4_ over 12 months.

**Conclusion:**

Detectable, significant molecular changes are observed in SF following KJD, that are remarkably consistent between individuals. Preliminary findings appear to suggest that increases in some molecules are associated with clinically meaningful responses. Joint distraction may provide a potential opportunity in the future to define regenerative biomarker(s) and identify pathways that drive intrinsic cartilage repair.

## Introduction

Osteoarthritis (OA) affects all joint tissues, with articular cartilage loss being one of the hallmarks of progressive disease^1^. It is likely that excessive mechanical load or loss of mechano-protective mechanisms in the joint is an underlying process in many cases of disease, but that there are other superimposed factors such as inflammation that modify its course^2^, ^3^, ^4^, ^5^, ^6^. Longitudinal cohorts such as the Osteoarthritis Initiative and Clinical Assessment of the Knee (CAS-K) show that in ∼40% of individuals with early knee OA, pain may stabilise or improve over time, suggesting that the disease may remit and is not inevitably progressive^7^. Interventions that mechanically off-load the joint, such as strengthening exercises, weight loss, orthotics such as bracing or surgical interventions such as osteotomy or unloading devices all reduce knee symptoms^1^^,^^8^.

It is often stated that adult articular cartilage is unable to repair but a body of literature is emerging that challenges this concept. This is best exemplified by traumatic focal cartilage defects that can repair spontaneously in young joints (reviewed in^9^), but in individuals undergoing high tibial or distal femoral osteotomy for OA, structural modification has also been observed^10^. The other evidence comes from studies of surgical knee joint distraction (KJD). The primary goal of this treatment is to improve symptoms sufficiently to delay knee arthroplasty. This is especially the case in younger patients, since these individuals have an increased risk of revision arthroplasty^11^. KJD is a technique where, under anaesthesia, an external fixation frame is placed on both sides of the joint, allowing distraction (gradual pulling apart of the joint's bony ends by ∼5 mm for 6 weeks). During distraction, the patient is encouraged to weight-bear on the extended knee. Such weight-bearing creates intermittent joint fluid pressure changes, due to built-in springs in the frame enabling a maximal 3 mm axial displacement under full body weight^12^. Studies of joint distraction have shown sustained and clinically significant improvement at a number of joint sites^13^^,^^14^. For knee OA, joint distraction improved knee symptoms for 5–9 years in individuals with established OA^15^^,^^16^. Remarkably, the 6 week intervention also led to apparent cartilage regeneration in the subsequent months and years, with increase in joint space width on X-ray, and increased articular cartilage thickness on magnetic resonance imaging (MRI)^14^^,^^16^, ^17^, ^18^. These studies suggest that, by temporarily off-loading the joint, KJD might somehow be responsible for ‘priming’ the joint to enable intrinsic cartilaginous repair. The biological mechanisms which underlie such a response are not understood but may include changes in the peri-articular bone and enhanced mesenchymal stem cell attachment to the damaged joint surface^19^^,^^20^. KJD is therefore an attractive mechanistic model in which to investigate potential reparative pathways and identify novel associated markers of clinical response.

Synovial fluid (SF) represents an accessible fluid that contains molecules reflecting biological processes within the joint. These molecules are joint tissue-agnostic; likely being derived from all the tissues interfacing the joint cavity and can be sampled repeatedly to monitor change over time within an individual. SF may represent joint tissue changes more accurately than measurements from blood or urine^21^^,^^22^. We have previously investigated 7 candidate proteins in the SF of individuals after acute knee injury. These molecules were originally shown to be induced in murine knee OA in a highly mechanosensitive manner^3^. 6 out of 7 proteins were found to be substantially up-regulated in those with acutely injured knees compared with controls^23^. These molecules included interleukin (IL)-6, matrix metalloproteinase (MMP)3 and monocyte chemoattractant protein (MCP)-1, associated with inflammatory activation but also others such as activin A, tumour necrosis factor-stimulated gene (TSG)-6 or tissue inhibitor of metalloproteinases (TIMP)-1, which have purported anti-catabolic/anabolic roles^24^. Our preclinical work has also identified candidate chondro-protective molecules that are released by damaged cartilage including FGF-2 and TGFβ^25^, ^26^, ^27^. Both of these are present in SF and have roles in chondrogenesis^28^^,^^29^.

We hypothesised that over the course of KJD, changes in the joint's mechanical environment modulate these candidate SF markers. We further hypothesised that changes in these mechanosensitive molecules either alone or in combination would be associated with clinical outcome. We set out to test these hypotheses in a proof-of-concept study in a group of individuals undergoing planned surgical KJD.

## Method

### Ethics

Approval for this study was given by a research ethics committee (#15-160/D; NL51539.041.15). Usual care clinical data was also accessed (#17-005). All participants gave written informed consent to participate prior to screening, according to the Declaration of Helsinki.

### Participants

Potential participants were identified by the orthopaedic surgeon (RC) from a population with knee OA attending for consideration of KJD as part of their usual clinical care at a single site in Netherlands (University Medical Center Utrecht). Inclusion criteria were: age<65 years; knee OA fulfilling ACR clinical criteria^30^; Kellgren and Lawrence (KL) grade≥2 on X-ray^31^; knee ligaments intact; preserved range-of-motion (flexion>120°; no loss of full extension); SF sample available at baseline. Exclusion criteria were: history of inflammatory arthritis affecting the index knee including rheumatoid arthritis; recent infection or systemic inflammatory disease; post-traumatic fibrosis; tibial plateau fracture; extensive bone-on-bone contact on X-ray; previous or planned knee arthroplasty during study period; surgery to the index knee within last 6 months; primary (isolated) patellofemoral OA; contralateral knee requiring surgical treatment; inability/contraindication/not consenting to provide SF; BMI≥35 kg/m^2^; pregnancy.

### Clinical outcomes

**Fig. 1:**
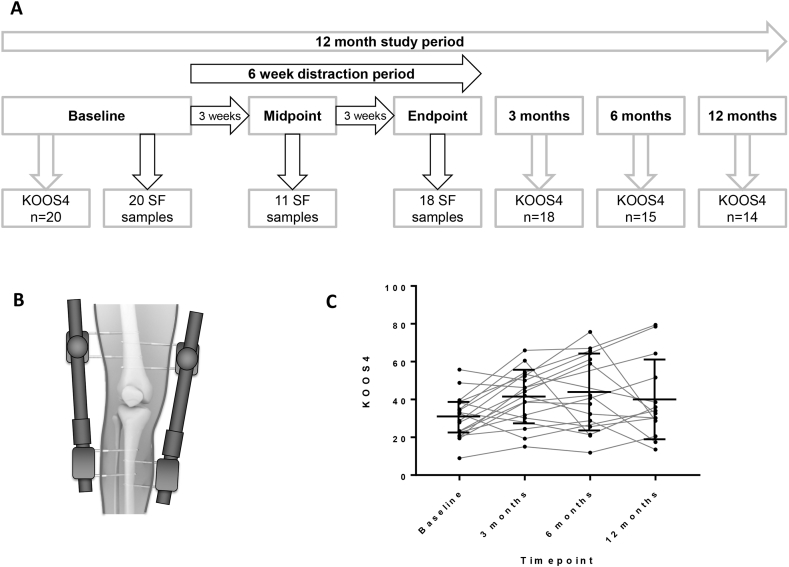
**Design and outcome measures of a proof-of-concept study to investigate synovial fluid analytes at time of knee joint distraction. A,** Flow chart indicating timings of study visits, collection of synovial fluid samples and collection of KOOS from 20 participants, including completeness of sampling/data over the 12 month study period. A further 2 participants gave consent but no baseline SF could be aspirated so they were excluded from further analysis as per protocol**. B**, Illustration of distraction frame which is surgically placed on the knee joint for a 6 week period. **C**, KOOS_4_ measurements in participants at baseline (pre-distraction), 3 months, 6 months and 12 months after surgical knee joint distraction. Medians and inter-quartile ranges are shown (bar and line). Abbreviations: KOOS, Knee Injury and Osteoarthritis Outcome Score (KOOS_4_ is composite measure of 4 domains); SF, synovial fluid.

Knee Injury and Osteoarthritis Outcome Score (KOOS) was collected as part of usual hospital care electronically at baseline, 3, 6 and 12 months [Fig. 1(A)]. From this, KOOS_4_, a single composite score which has been validated as a single outcome in other clinical studies was calculated (the mean of 4 of 5 KOOS subscales: Pain, Symptoms, Sports/Recreation and Quality of Life)^32^^,^^33^.

### Usual care intervention

A non-hinged, external proof-of-concept fixation joint distraction frame (Monotube Triax with pin clamps, Stryker) [Fig. 1(B)] was fitted to the index knee by an orthopaedic surgeon (RC) whilst the patient was under spinal or general anaesthesia (GA) and the joint surfaces distracted by 5 mm. The frame was then worn for 6–7 weeks.

### Participant biological samples

A maximum of 2 ml of SF was aspirated by needle from the index knee at baseline visit (whilst participant under anaesthesia and prior to the distraction frame being fitted), subsequently at midpoint of distraction (3-4 weeks, under local anaesthesia) and at endpoint of distraction (at 6-7 weeks, immediately after the distraction frame was removed under anaesthesia) [Fig. 1(A)]. Within 2 h, all samples were centrifuged for 20 min at 3000G. Supernatants were stored in 200 μl aliquots in cryovials at −80°C in monitored freezers.

### Comparator ranges

*Normal:* These were calculated in previously collected SF from patients undergoing amputation for treatment of lower limb tumour, at Royal National Orthopaedic Hospital (Stanmore), London, UK, or transplant donation, at Charing Cross Hospital, London, UK (REC 09/H0710/60), who had macroscopically normal knee articular cartilage at the time of surgery and no evidence of arthritis or tumour invasion into the joint^23^. *OA:* These were calculated from measurements in SF from research tissue bank samples of patients with a confirmed diagnosis of OA undergoing partial or total joint replacement surgery at the Nuffield Orthopaedic Centre, Oxford, UK (REC 09/H0606/11 + 5). SF had been processed and stored as above.

### Reagents

General laboratory reagents were the best available grade from either Sigma–Aldrich (Dorset, UK) or BDH (Dorset, UK) unless otherwise stated. MesoScale Discovery (MSD) plates and MSD SULFO-TAG labelled Streptavidin (#R32AD-5) were from MSD (Rockville, MD, USA). Enzyme-linked immunosorbent assays (ELISAs) were from commercial providers (Table I).

**Table I:** Assay characteristics of panel of 10 candidate markers

Analyte	Assay	Manufacturer (Catalog No.)	Intra-Assay CV	Inter-Assay CV	Lower Limit of Normal (SF)	Upper Limit of Normal (SF)	Dil_n_ Factor (SF)
			(%)	(%)	(pg/ml)	(pg/ml)	
Activin A	Human/Mouse/Rat Activin A Quantikine ELISA	R&D (DAC00B)	3.9	11.7	1,028	5,253	50
MCP-1	V-PLEX Human MCP-1	MSD (K151NND-1)	3.1	5.1	60	493	5
FGF-2	V-PLEX Human (basic) FGF-2	MSD (K151MDD-1)	4.0	6.1	2	411	4
IL-6	V-PLEX Custom Human Cytokine	MSD (K151A0H-1)	4.1	9.7	1	20	5
IL-8	V-PLEX Custom Human Cytokine	MSD (K151A0H-1)	3.5	8.9	2	39	5
LTBP2	Human LTBP2 ELISA	Abbexa (abx 152242)	6.6	17.3	1,887	13,630	4
MMP3	Human MMP3 Ultra-Sensitive	MSD (K151FZC-1)	4.7	13.6	3,742	231,000	50
TGFβ-1	Human TGFβ-1 Quantikine ELISA	R&D (DB100B)	3.7	13.0	257.3	1,545	4
TIMP-1	Human TIMP-1 Ultra-sensitive	MSD (K151JFC-1)	9.1	10.3	143,000	744,700	200
TSG-6	In-house, self-coated MSD	MSD (L15XA-1)	4.5	7.1	6,479	19,060	6

Inter- and intra-assay coefficients of variation (C.V.s) were calculated for all assays. Lower and upper limits were also calculated for all assays for normal ranges using the geometric mean ± 2 standard deviations.

Abbreviations: CV -coefficient of variation; Dil_n_ -Dilution; MSD Mesoscale Discovery.

### Assays

Assays were conducted for 10 pre-defined candidate molecules listed in Table I. All assays were carried out as per manufacturers’ instructions unless stated otherwise. Each assay had either previously undergone validation by us^23^ or else underwent structured performance assessment and optimisation for SF for this project, and all also passed quality performance requirements during sample reads (Table I). ELISA plates were read using Berthold Mithras LB940 reader and MSD plates by MSD QuickPlex SQ120 reader (analysed with MSD Discovery Workbench software v4.0.12). For TSG-6, each plate well (MSD, Rockville, USA, L15XA) was custom-coated with 30 μl 10 μg/ml TSG-6 capture antibody (Merck, MABT108) in phosphate-buffered saline (PBS) overnight at 4°C. Methods were then as described^23^. Mean concentrations of analytes were calculated from duplicate assay reads for each participant for each timepoint. Inter- and intra-assay coefficients of variation (C.V.s) were calculated for all assays. The lower limit of quantitation (LLOQ) was calculated for all assays. Where a measurement was below LLOQ, 50% of this value was used^22^^,^^23^. Lower and upper limits were also calculated for all assays for normal ranges using the geometric mean ± 2 standard deviations.

### Statistical analysis

All available data were analysed on all participants with sufficient SF at each of the 3 timepoints (and one patient with samples at baseline and 3 weeks). All SF analytes were above LLOQ (allowing attribution of endpoint measurements) except for one sample each for TGFβ1 at baseline, FGF-2 at midpoint and activin A at endpoint. These values were considered as 50% of the LLOQ. Sample and KOOS completeness are shown in Fig. 1(A). Missing data were not imputed.

#### Change in KOOS_4_ over time

Median differences between paired observations of KOOS_4_ at baseline and either 3, 6 or 12 months were compared by Wilcoxon signed rank test.

#### Change in analyte levels over time

Median differences between paired observations (baseline vs 3 or 6 weeks) of analyte levels were compared by Wilcoxon signed rank test. Effect size (ES) was reported as the difference between medians. Correlations between the changes over 6 weeks for each analyte were assessed by Spearman's R coefficient (range −1 to 1; where ±1 = strongest positive (or negative) correlation, 0 = no correlation).

#### Association of change in analytes with KOOS_4_

The clinical outcome variable was change in KOOS_4_ over time (KOOS_4_ at either 3, 6 or 12 months respectively−KOOS_4_ at baseline). Linear regression was employed to model the relationship between continuous change in analyte levels (concentrations at 6 or 3 weeks – baseline concentrations) and change in KOOS_4_.

In a planned secondary analysis, linear regression also assessed change in KOOS_4_ by categories of change in analytes. Concentrations of analytes (at baseline and 6 weeks) were classified into normal (≥25th and <75th percentiles), high (≥75th percentile) and low (<25th percentile) categories. The 25th and 75th centiles were calculated from measurements of these molecules in SF from 40 individuals with OA who had undergone either partial or total knee joint replacement (see Comparator ranges). These data were generated at same time as participant data, using the same assay batches. ‘Relevant change’ was defined as a movement between at least one category from baseline to 6 weeks (relevant increase, or relevant decrease), or as no relevant change.

#### Change in analytes by responders and non-responders in KOOS_4_

Responders (those whose change in KOOS_4_ over 6 months (the latest point at which there was clinical change from baseline), [Fig. 1(C)] was ≥10 points, i.e., the minimal clinically important difference (MICD) for KOOS_4_)^32^; and Non-Responders (those whose KOOS_4_ change over 6 months was <10 points) were categorized. Differences between molecular changes in these 2 groups were compared by Mann-Whitney *U* test.

Data were stored on a secure database (OpenClinica). Analysis was performed in STATA IC 13.1 and Graphpad Prism 6.03.

## Results

13/20 (65%) participants were male with mean age 55 ± 5 years (Table II). All had KL grade≥2; 18 (90%) grade 3/4, with substantial knee pain at baseline (KOOS pain 38.6 ± 16.0; where 100 is no pain, normal function). As expected from previously published studies, there was an improvement in KOOS_4_ in the subsequent months following the intervention [Fig. 1(C)].

**Table II:** Baseline characteristics of study participants

Baseline Characteristic	N (%), or mean, SD
**Sex**	
Male	13 (65%)
Female	7 (35%)
**Age (years)**	55, 5
**Body Mass Index (BMI)**	29, 3
**Kellgren and Lawrence grade**	
2	2 (10%)
3	10 (50%)
4	8 (40%)
**KOOS**_**4**_	30, 11

Abbreviations: SD Standard deviation; KOOS_4_, Knee injury and OA outcome score-4.

**Fig. 2:**
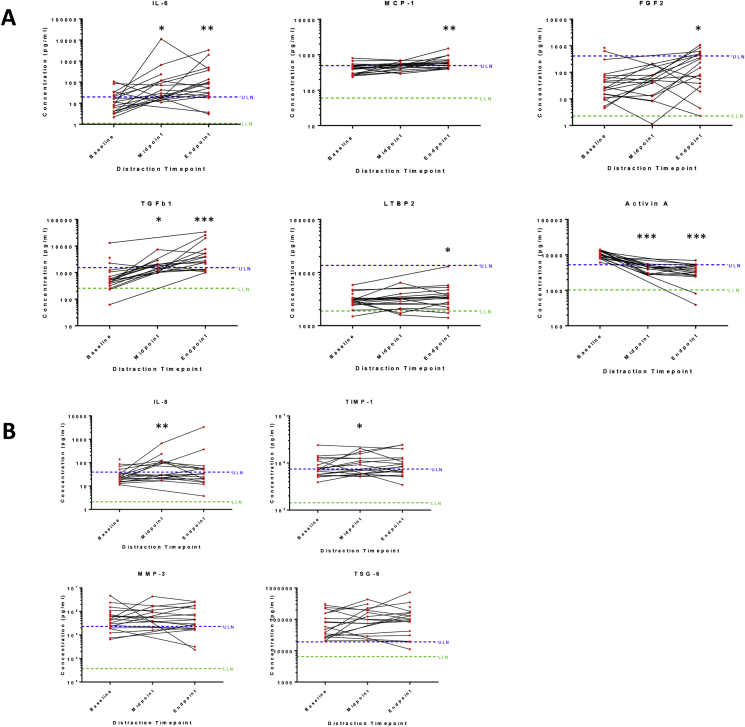
**Measurement of synovial fluid analytes during knee joint distraction.** Synovial fluid from study participants immediately prior to distraction (‘baseline’), after 3 weeks of knee joint distraction (‘midpoint’) and at 6 weeks after knee joint distraction (‘endpoint’) were assayed for pre-defined markers of interest by electrochemiluminescence or ELISA (see Table I). Measurements for each of 10 analytes are shown, with mean concentrations for each analyte plotted on a log 10 *y* axis. *P < 0.05,**P < 0.01,***P < 0.001 by Wilcoxon signed rank test, comparing paired levels at end point or midpoint vs baseline (individual *P* values are given in Supplementary Table 1). **A,** shows 6 analytes with change at endpoint vs baseline. B, shows 4 analytes without change at endpoint (although upper 2 showed change at midpoint). ULN and LLN of normal ranges were calculated for each analyte as described in methods and Table I. Abbreviations: LLOQ, lower limit of quantification; ULN, upper limit of normal; LLN, lower limit of normal; LTBP2, latent-transforming growth factor beta-binding protein 2; TGFβ-1, transforming growth factor beta 1; FGF-2, basic fibroblast growth factor; TIMP-1, tissue inhibitor of metalloproteinases 1; TSG-6, tumour necrosis factor-inducible gene 6 protein; IL-6, interleukin 6; MCP-1, monocyte chemoattractant protein 1; IL-8, interleukin 8; MMP3, matrix metalloproteinase-3.

6/10 SF analytes showed changes between baseline and 6 weeks (IL-6, ES = 56.6, *P* = 0.0043; MCP-1, ES = 155.1, *P* = 0.0016; FGF-2, ES = 164.7, *P* = 0.0123; TGFβ-1, ES = 2.1, *P* = 0.0003; LTBP2, ES = 0.4, *P* = 0.0475; activin A, ES = −6.8, *P* = 0.0002) [Fig. 2(A)] and (Supplementary Table 1). Of these, IL-6, MCP-1, FGF-2 and TGFβ-1 showed a predominant increase in levels, while activin A mainly decreased (to within normal range for most individuals). There was variation in response between individuals, exemplified by LTBP-2. For several analytes, change was detectable within 3 weeks of distraction (activin A, TGFβ-1 and IL-6) (Supplementary Table 1). 2 further molecules, IL-8 and TIMP-1 were different at 3 weeks (ES = 73.5 and ES = 389, respectively), but not at 6 weeks (ES = 10.5, ES = 115.2, respectively) [Fig. 2(B)], (upper panels). The remaining 2 analytes (MMP3, TSG-6) did not change over the distraction period (ES = −75.3, *P* = 0.53 and ES = 41,508, *P* = 0.21 respectively, Fig. 2(B), lower panels).

**Fig. 3:**
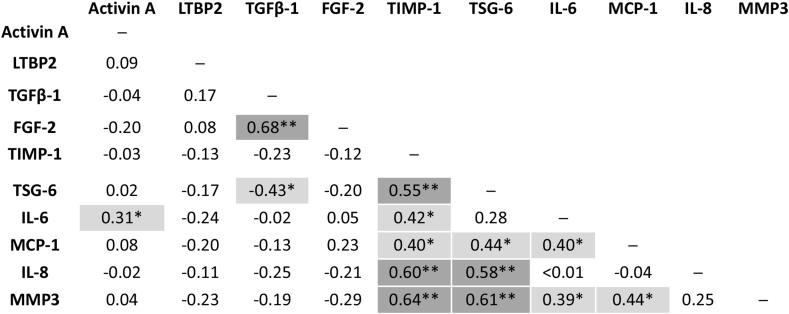
**Correlation between change of analytes in the synovial fluid of participants over period of knee joint distraction.** Spearman rank tests were performed to determine correlations between the change in levels of synovial fluid analytes over the 6 week distraction period (concentration at 6 weeks-baseline concentrations). Correlation coefficients were calculated using all available participant data and the mean of 2 repeated (duplicate) measures for each synovial fluid sample. Strength of correlation by Spearman R coefficient is shown: * (Mid grey shading): Low positive (negative) correlation, 0.30 to 0.49 (−0.30 to −0.49). ** (Dark grey shading): Moderate positive (negative) correlation, 0.50 to 0.69 (−0.50 to −0.69). Abbreviations: LTBP2, latent-transforming growth factor beta-binding protein 2; TGFβ-1, transforming growth factor beta 1; FGF-2, basic fibroblast growth factor; TIMP-1, tissue inhibitor of metalloproteinases 1; TSG-6, tumour necrosis factor-inducible gene 6 protein; IL-6, interleukin 6; MCP-1, monocyte chemoattractant protein 1; IL-8, interleukin 8; MMP3, matrix metalloproteinase 3.

Several analytes correlated with each other in their change over the 6 week distraction period (Fig. 3). Associations between changes in markers could also be seen over the initial 3 weeks of knee joint distraction (Supplementary Figure 1). Higher correlations were found for TGFβ-1 and FGF-2 (*R* = 0.68); IL-6, TIMP-1 and either MMP3 or TSG-6; (all pairs *R* > 0.5). LTBP2 and activin A had low correlation with other analytes over time. TGFβ-1 and IL-6 were negatively correlated (*R* = −0.43).

**Fig. 4:**
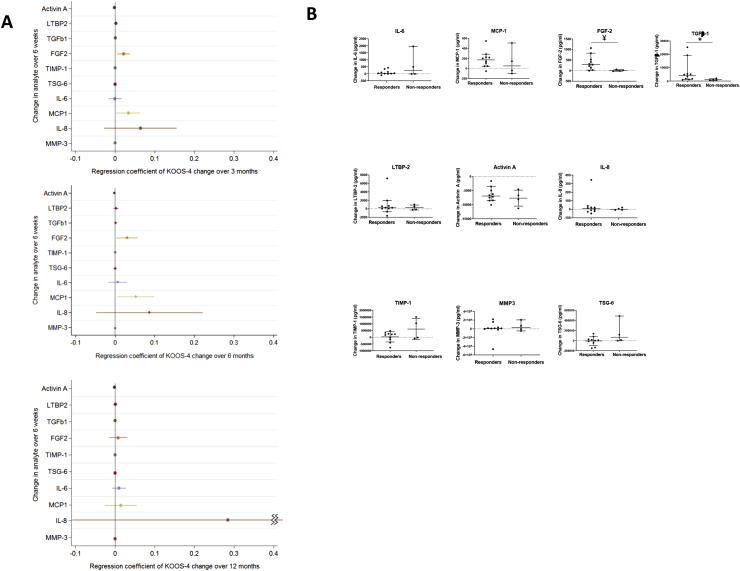
**Association of change in synovial fluid analytes with the clinical outcome KOOS**_**4.**_. **A** Linear regression models for the association of the change over the distraction period for each of 10 synovial fluid analytes (measured in pg/ml) with participants' change in KOOS_4_ over varying periods are shown: upper panel**,** change in KOOS_4_ over 3 months; middle panel, change in KOOS_4_ over 6 months and lower panel, change in KOOS_4_ over 12 months. Forest Plots of unadjusted (crude) results, including the regression coefficient for the effect on KOOS_4_ change over the specified period and 95% confidence intervals are shown (data also shown in Supplementary Table 2). **B** The change of concentration in each analyte over the 6 week distraction period is shown (6 week level – baseline level), for 2 subgroups: Responders (those whose change in KOOS_4_ over 6 months was ≥10 points, i.e., those achieving the MICD for KOOS_4_); and Non-Responders (those whose change in KOOS_4_ over 6 months was <10 points, i.e., those not achieving the MCID for KOOS_4_). The bars represent the median and 95% Confidence Intervals for each group. Between group comparisons were by Mann-Whitney U test, *P = 0.04; ¥*P* = 0.01. Abbreviations: MCID, minimal clinically important difference; LTBP2, latent-transforming growth factor beta-binding protein 2; TGFβ-1, transforming growth factor beta 1; FGF-2, basic fibroblast growth factor; TIMP-1, tissue inhibitor of metalloproteinases 1; TSG-6, tumour necrosis factor-inducible gene 6 protein; IL-6, interleukin 6; MCP-1, monocyte chemoattractant protein 1; IL-8, interleukin 8; MMP3, matrix metalloproteinase-3; KOOS, Knee Injury and Osteoarthritis Outcome Score (KOOS_4_ is composite measure of 4 domains).

The association of change in candidate molecules over the distraction period with subsequent change in KOOS_4_ was examined. Change in 4 molecules was associated with change in KOOS_4_ over the first 3 months: activin A, TGFβ-1, FGF-2 and MCP-1 [Fig. 4(A)]. For all except activin A, an increase in the analyte was associated with greater improvement in KOOS_4_, but the effects were weak (Supplementary Table 2). The low effect sizes were primarily because the unit of change of a marker within the regression model was per 1 pg/ml, whereas often much larger changes in markers than 1 pg/ml were seen. Similar associations persisted at 6 months for all 4 molecules. For example, for the effect of change in FGF-2 over 6-weeks, on change in KOOS_4_ over 6-months, for a 1-unit increase in FGF-2 change per pg/ml, the change in KOOS_4_ is 0.03 points. To interpret the 95% CI, the underlying effect in the population could lie between 0.004 and 0.057. To aid interpretation, the median increase of FGF-2 over 6 weeks is 165 pg/ml. Hence for a 165 pg/ml unit increase in FGF-2, the change in KOOS_4_ is 4.95 points (95%CI 0.66 to 9.41). IL-8 had the largest and increasing effect size (0.28 by 12 months), but the confidence intervals at all timepoints were wide.

To test the relevance of these findings, we categorised participants’ molecular measurements as having no relevant change, a relevant increase or a relevant decrease over the 6 week distraction period (see methods) and examined the association of these categories with change in KOOS_4_. Those with a relevant increase in SF IL-8 during the distraction period had a greater improvement in KOOS_4_ over 12 months than those with no change (regression coefficient 17.6 [1.2, 34.0]; *P* = 0.04). However, no other molecular changes were associated with clinical outcome when categorised in this way (Supplementary Table 3). Furthermore, the confidence intervals for this observation are wide and given that the other findings for IL-8 did not reach significance [Fig. 4(A)], this could be a chance finding).

We also compared the change in analyte levels over the 6 weeks of distraction in those making the MCID of 10 points or more by KOOS_4_ (responders) with those who did not improve by this amount (non-responders). The clinical response to joint distraction was most pronounced at 6 months, with 11/15 (73%) of individuals with available data reaching a MCID on KOOS_4_. Responders at 6 months had a greater increase in TGFβ-1 and FGF-2 during the distraction period than non-responders [Fig. 4(B)], (Supplementary Table 4 and Supplementary Figure 2). Similar analyte changes were also seen in responders and non-responders at 3 months, when TIMP-1 levels were also different between the 2 groups (ES = 497 ng/ml, *P* = 0.02, Supplementary Figure 3).

## Discussion

Easily detectable, substantial changes in levels of 8 putative mechanosensitive molecules of the inflammatory response (activin A, LTBP2, TGFβ-1, FGF-2, TIMP-1, IL-6, MCP-1, and IL-8) were seen in SF over the period of KJD. There were also associations between several of these molecules over time. These changes would not appear to be due to SF volume change because whilst some analytes increase, others stay the same or even decrease. Of the regulated molecules, whilst IL-6 and MCP-1 (also known as CCL-2) have been associated with degeneration or pain in the osteoarthritic joint^34^^,^^35^, FGF-2 and TGFβ-1 are more typically associated with repair^27^^,^^36^. It is perhaps not surprising that a mechanically-induced inflammatory response should include both catabolic and reparative processes. But that an intervention which apparently leads to net articular cartilage repair involves the activation of traditionally inflammatory pathways would go against current convention. Overall there was substantial variation between individuals for certain molecules in the extent and sometimes direction of this response. This supports the notion that an individual's biological response to the intervention could vary and be related to their clinical response. On the other hand, some molecules like TGFβ-1 and Activin A showed very consistent directional changes following KJD.

Our proof-of-concept study appears to suggest an association between this measurable biological response to joint distraction and subsequent clinical outcome. The clinical response to joint distraction was most pronounced at 6 months. Several of the associations between change in analytes and KOOS_4_ at 6 months were also apparent at 3 and 12 months, and when individuals were stratified, either by their molecular response or their clinical response. This supports that elements of this biological response to distraction appeared to be associated with a clinically meaningful response: for example, FGF-2 and TGFβ-1, typically associated with cartilage anabolism/anti-catabolism, were raised in responders^37^^,^^38^.

One molecule, activin A, strikingly fell in all individuals to what we estimate are normal levels in human SF. Activin A is produced by osteoarthritic and injured articular cartilage and promotes skin wound healing^39^^,^^40^. Its direction of change (opposite to that of FGF-2/TGFβ-1) is perhaps surprising: activin A is a TGFβ superfamily member^24^ and is strongly FGF-2-dependent in the joint in our pre-clinical studies^38^^,^^39^. It may be that these apparent paradoxes are because different joint tissues are involved: FGF-2 and TGFβ may derive from the capsule, say, whereas joint-offloading may reduce the cartilage injury response, reducing activin A. Whilst activin A appears to be a highly sensitive read-out of the intervention, it does not show association with benefit, perhaps because its change is so consistent.

There are some limitations of this study. It is important to avoid over-interpretation of what was a small experimental study, and we have reported confidence intervals of association of change in molecules with change in KOOS_4_, to reflect the level of certainty. No correction was made for multiple testing: some apparent associations could have been found by chance.

Identification of truly ‘normal’ individuals or those with OA who are of exactly the same demographic and stage of disease and who are willing to undergo sampling of SF is challenging. Our ‘Normal comparator’ group included those individuals undergoing surgery for musculoskeletal tumours, which could potentially have influenced circulating levels of analytes. The OA comparator group was selected from a bank of samples collected at the time of arthroplasty to be as similar as possible in terms of gender and age. However, it is possible by the nature of the intervention that some would have had more advanced stage of disease or slight differences in other factors to those in the Joint Distraction group, meaning they were not directly comparable. However, the validity of our findings do not rely solely on such Comparator data (with most comparisons made within the cohort of those undergoing distraction).

These results need to be validated in a larger, independent study, where potentially relevant additional covariates (such as age, BMI) and structural imaging outcomes are incorporated^13^ (Correlation of marker change with imaging-based measures of cartilage thickness or volume in this current small study was not included as there would have been lack of power to detect an effect). As this intervention was part of usual care and not a clinical trial, this may have led to increased missing data and somewhat less striking improvement in clinical outcomes. This contrasts with previously published data in KJD suggesting a clinically significant long-term response^14^^,^^16^, ^17^, ^18^. A comparison between patients treated in clinical practice and those in (randomised) clinical trials revealed no clear differences in clinical outcomes between these 2 settings at 12 months (data to be published elsewhere). This study was not designed to detect a difference in clinical outcome and likely lacked power to detect differences at 12 months.

In summary, we have shown a measurable molecular response in SF to joint distraction, which appears to be associated with patient-reported outcomes. This observation supports the accurate measurement of this response in SF as both possible and informative^22^^,^^41^. Ways of finding associations with a positive outcome to distraction are currently limited^13^. These observations show the potential to define biomarker(s) associated with positive clinical responses to this and similar interventions aiming to off-load the joint surfaces^42^^,^^43^. Biomarker stratification, identifying individuals most likely to respond in clinical trials or usual care would increase the utility of this already apparently cost-effective intervention^44^. Experimental studies of joint distraction represent a novel way of identifying potential regenerative pathways that drive intrinsic connective tissue repair; these pathways might be amenable to augmentation, by pharmacological or other means to treat symptomatic OA.

## Studies involving humans/animals

The procedures followed were in accordance with the ethical standards of the responsible committees on human experimentation (institutional and national) and with the Helsinki Declaration of 1975, as revised in 2000.

## Data availability

Data generated by this research will be made available on reasonable request to the authors where this is legally and ethically possible.

## Author contributions

All authors fulfilled the ICMJE criteria below.

All authors have made substantial contributions to all three of sections(1), (2) and (3) below:

(1) the conception and design of the study, or acquisition of data, or analysis and interpretation of data
(2) drafting the article or revising it critically for important intellectual content
(3) final approval of the version to be submitted

Specifically.

Planning and design of study TV, FW, SM, FL, RC, CG, AJ.

Conduct, data collection and biomarker analysis MJ, RC, SM, FW, RH, BH.

Statistical analysis and reporting CG, AJ, BH, FW, TV, SM, RC, FL.

Fiona Watt (Fiona.watt@kennedy.ox.ac.uk), Tonia Vincent (tonia.vincent@kennedy.ox.ac.uk) and Simon Mastbergen (s.mastbergen@umcutrecht.nl) take joint responsibility for the integrity of the work as a whole, from inception to finished article.

## Conflict of interest

Fiona Watt: no conflicts of interest; other unrelated funding received from Pfizer Ltd, USA clinical study (AZR00860) and from Astellas European Foundation clinical study (AZR00850).

Benjamin Hamid: no conflicts of interest.

Cesar Garriga: no conflicts of interest.

Andrew Judge: no conflicts of interest.

Renata Hrusecka: no conflicts of interest.

Roel Custers: no conflicts of interest.

Mylene Jansen: no conflicts of interest.

Floris Lafeber - is co-founder, shareholder, and co-director of ArthroSave BV, and is consultant for Synerkine Pharma BV, both spin-off companies of the UMC Utrecht involved in treatment of osteoarthritis.

Simon Mastbergen: no conflicts of interest.

Tonia Vincent: no conflicts of interest.

## Role of funding source

This work was supported by coordinated project grants from Versus Arthritis project grant (20783), Centre for OA Pathogenesis Versus Arthritis (grants 20205 and 21621) and the Kennedy Trust for Rheumatology Research (all of the UK), and also ReumaNederland, the Dutch Arthritis Society (ISP14-3-301/16-1-404) for the work in the UK and The Netherlands for the study respectively. FEW is supported by a UKRI Future Leaders Fellowship and was supported during this work in part by the NIHR Oxford Biomedical Research Centre. The views expressed are those of the authors and not necessarily those of the NHS, the NIHR or the Department of Health.

The study funders/study sponsors had no involvement in the study design, data collection, analysis or interpretation of the study, or in the writing of, or decision to submit the manuscript.
