# Supplementary material for: The molecular profile of synovial fluid changes upon joint distraction and is associated with clinical response in knee osteoarthritis
Source: Osteoarthritis Cartilage. 2020 Mar;28(3):324–33. doi: 10.1016/j.joca.2019.12.005 (PMC7054834; doi:10.1016/j.joca.2019.12.005)
Supplement: Multimedia component 1 [file mmc1.docx]

| **Analyte** | **Baseline**  **(median, IQR)** | **3 weeks**  **(median, IQR)** | **Effect size** | **P value** | **6 weeks**  **(median, IQR)** | **Effect size** | **P value** |
| --- | --- | --- | --- | --- | --- | --- | --- |
| Activin A (ng/ml) | 10.5  (8.1, 12.9) | 4.0  (3.0, 5.0) | -6.5 | 0.003 | 3.7  (2.0, 5.4) | -6.8 | 0.0002 |
| LTBP2 (ng/ml) | 3.0  (2.7, 3.3) | 3.1  (2.0, 3.2) | 0.1 | 0.37 | 3.4  (2.5, 4.0) | 0.4 | 0.05 |
| TGFβ-1 (ng/ml) | 0.5  (0.4, 1.1) | 2.0  (1.1, 2.1) | 1.5 | 0.01 | 2.6  (1.3, 5.3) | 2.1 | 0.0003 |
| FGF-2 (pg/ml) | 26.6  (13.0, 69.3) | 39.3  (8.6, 77.4) | 12.7 | 0.42 | 191.3  (38.4, 496.1) | 164.7 | 0.01 |
| TIMP-1 (ng/ml) | 729.0  (563.1, 1,163.5) | 1,118.0  (717.5, 1,568.0) | 389 | 0.02 | 844.2  (624.3, 1,225.0) | 115.2 | 0.11 |
| TSG-6 (ng/ml) | 58.5  (28.5, 156.9) | 122.4  (73.7, 227.4) | 63.9 | 0.29 | 100.0  (42.4, 164.3) | 41.5 | 0.21 |
| IL-6 (pg/ml) | 11.3  (4.2, 28.4) | 70.9  (23.2, 236.7) | 59.6 | 0.02 | 67.9  (23.2, 366.2) | 56.6 | 0.004 |
| MCP-1 (pg/ml) | 410.0  (282.9, 482.6) | 524.8  (485.5, 658.1) | 114.8 | 0.25 | 565.1  (440.1, 681.4) | 155.1 | 0.002 |
| IL-8 (pg/ml) | 23.1  (16.0, 35.3) | 96.6  (28.7, 122.6) | 73.5 | 0.01 | 33.6  (19.8, 56.4) | 10.5 | 0.31 |
| MMP3 (ng/ml) | 444.3  (215.7, 815.0) | 912.2  (358.2, 1,623.3) | 467.9 | 0.86 | 369.0  (190.9, 1,361.8) | -75.3 | 0.53 |

**Supplementary Table 1.** Biomarker levels at baseline, 3 weeks and 6 weeks post knee joint distraction. Comparison was by Wilcoxon signed rank test, comparing mean of duplicate measures at 3 weeks or 6 weeks to measures at baseline. Data are also shown graphically in Figure 2 (please note some units are shown in ng/ml rather than pg/ml in this table). Effect size is calculated as the difference of medians.

IQR, interquartile range; LTBP2, latent-transforming growth factor beta-binding protein 2; TGFβ-1, transforming growth factor beta 1; FGF-2, basic fibroblast growth factor; TIMP-1, tissue inhibitor of metalloproteinases 1; TSG-6, TNF stimulated protein-6; IL-6, interleukin 6; MCP-1, monocyte chemoattractant protein 1; IL-8, interleukin 8; MMP3, matrix metalloproteinase-3.

| **Change of KOOS_4_ over defined period** | **Change in analyte** | **Coefficient** | **95% confidence interval** | **R squared** | **P value** |
| --- | --- | --- | --- | --- | --- |
| 3 months | Activin A | -0.001 | -0.002, <0.001 | 0.39 | 0.01 |
|  | LTBP2 | 0.002 | -0.002, 0.006 | 0.07 | 0.29 |
|  | TGFβ-1 | 0.001 | <0.001, 0.002 | 0.37 | 0.01 |
|  | FGF-2 | 0.021 | 0.005, 0.038 | 0.34 | 0.01 |
|  | TIMP-1 | <0.001 | <0.001, <0.001 | <0.01 | 0.96 |
|  | TSG-6 | <0.001 | <0.001, <0.001 | <0.01 | 0.89 |
|  | IL-6 | <0.001 | -0.017, 0.016 | <0.01 | 0.96 |
|  | MCP-1 | 0.034 | 0.003, 0.064 | 0.27 | 0.03 |
|  | IL-8 | 0.064 | -0.028, 0.156 | 0.13 | 0.16 |
|  | MMP3 | <0.001 | <0.001, <0.001 | 0.05 | 0.41 |
| 6 months | Activin A | -0.002 | -0.003, <0.001 | 0.36 | 0.02 |
|  | LTBP2 | 0.002 | -0.004, 0.009 | 0.05 | 0.42 |
|  | TGFβ-1 | 0.002 | <0.001, 0.003 | 0.35 | 0.02 |
|  | FGF-2 | 0.030 | 0.004, 0.057 | 0.32 | 0.03 |
|  | TIMP-1 | <0.001 | <0.001, <0.001 | <0.01 | 0.92 |
|  | TSG-6 | <0.001 | <0.001, <0.001 | 0.03 | 0.55 |
|  | IL-6 | 0.007 | -0.017, 0.031 | 0.03 | 0.55 |
|  | MCP-1 | 0.052 | -0.005, 0.098 | 0.31 | 0.03 |
|  | IL-8 | 0.087 | -0.048, 0.221 | 0.13 | 0.19 |
|  | MMP3 | <0.001 | <0.001, <0.001 | <0.01 | 0.81 |
| 12 months | Activin A | -0.002 | -0.006, 0.002 | 0.08 | 0.34 |
|  | LTBP2 | -0.002 | -0.007, 0.003 | 0.05 | 0.45 |
|  | TGFβ-1 | -0.001 | -0.002, 0.001 | 0.05 | 0.48 |
|  | FGF-2 | -0.001 | -0.025, 0.024 | <0.01 | 0.95 |
|  | TIMP-1 | <0.001 | <0.001, <0.001 | 0.02 | 0.62 |
|  | TSG-6 | <0.001 | <0.001, <0.001 | 0.05 | 0.45 |
|  | IL-6 | 0.005 | -0.014, 0.023 | 0.03 | 0.59 |
|  | MCP-1 | -0.013 | -0.062, 0.036 | 0.03 | 0.58 |
|  | IL-8 | 0.277 | -0.096, 0.651 | 0.20 | 0.13 |
|  | MMP3 | <0.001 | <0.001, <0.001 | 0.02 | 0.61 |

**Supplementary Table 2.** Regression coefficients and confidence intervals of the association of change in markers over distraction period with change in KOOS_4_ over 3 months, 6 months and 12 months (data are also shown by forest plot in Figure 4A).

|  |  | | |
| --- | --- | --- | --- |
| **Analyte (reference category)** | **Coefficient** | **95% CI** | **P value (Wald test)** |
| **Activin A (no change)** |  |  |  |
| decrease | 9.99 | (-6.03 to 26.02) | 0.20 |
| **LTBP2 (no change)** |  |  |  |
| increase | 8.16 | (-18.57 to 34.89) | 0.52 |
| **TGFβ-1 (no change)** |  |  |  |
| increase | 6.62 | (-5.72 to 18.96) | 0.26 |
| **FGF-2 (no change)** |  |  |  |
| increase | 5.52 | (-10.26 to 21.30) | 0.46 |
| decrease | 9.24 | (-18.08 to 36.57) | 0.47 |
| **TIMP-1 (no-change)** |  |  |  |
| increase | 11.38 | (-5.05 to 27.81) | 0.16 |
| decrease | -5.68 | (-42.42 to 31.06) | 0.74 |
| **IL-6 (no change)** |  |  |  |
| increase | 7.76 | (-4.73 to 20.24) | 0.20 |
| decrease | 2.95 | (-23.53 to 29.44) | 0.81 |
| **MCP-1 (no change)** |  |  |  |
| increase | 10.41 | (-4.55 to 25.36) | 0.15 |
| decrease | 6.90 | (-29.73 to 43.54) | 0.69 |
| **IL-8 (no change)** |  |  |  |
| increase | **17.63** | **(1.22 to 34.04)** | **0.04** |
| decrease | -4.32 | (-23.27 to 14.63) | 0.63 |
| **MMP3 (no-change)** |  |  |  |
| increase | 5.14 | (-18.06 to 28.33) | 0.64 |
| decrease | 0.61 | (-27.80 to 29.02) | 0.96 |

**Supplementary Table 3.** Change of KOOS_4_ over 12 months according to the response of biomarkers to knee joint distraction by categories of relevant change (decrease, no change, or increase) over 6 weeks (see Methods). N=15. CI, confidence interval; LTBP2, latent-transforming growth factor beta-binding protein 2; TGFβ-1, transforming growth factor beta 1; FGF-2, basic fibroblast growth factor; TIMP-1, tissue inhibitor of metalloproteinases 1; TSG-6, TNF stimulated protein-6; IL-6, interleukin 6; MCP-1, monocyte chemoattractant protein 1; IL-8, interleukin 8; MMP3, matrix metalloproteinase-3.

| **Analyte** | **Change in analyte in Responders**  **(median)** | **Change in analyte in Non-Responders**  **(median)** | **Effect size** | **P value** |
| --- | --- | --- | --- | --- |
| Activin A (ng/ml) | -6940 | -7443 | -503.2 | 0.64 |
| LTBP2 (ng/ml) | 270.0 | 207.4 | -62.7 | 0.84 |
| TGFβ-1 (ng/ml) | 4128 | 918 | -3210 | **0.036** |
| FGF-2 (pg/ml) | 297.1 | 1.23 | -295.9 | **0.014** |
| TIMP-1 (ng/ml) | 194.5 | 508.7 | 314.2 | 0.37 |
| TSG-6 (ng/ml) | 2.5 | 65.0 | 62.5 | 0.24 |
| IL-6 (pg/ml) | 33.87 | 241.7 | 207.9 | 0.84 |
| MCP-1 (pg/ml) | 170.1 | 50.78 | -119.3 | 0.54 |
| IL-8 (pg/ml) | 7.105 | -0.19 | -7.3 | 0.99 |
| MMP3 (ng/ml) | 102.3 | 315.8 | 213.5 | 0.99 |

**Supplementary Table 4 (supporting Figure 4B).** The median change in concentration of each analyte over the 6 week distraction period is shown (6 week level – baseline level), for 2 subgroups: Responders (those whose change in KOOS_4_ over 6 months was ≥ 10 points, i.e. those achieving the MICD for KOOS_4_); and Non-Responders (those whose change in KOOS_4_ over 6 months was <10 points, i.e. those not achieving the MCID for KOOS_4_). The calculated effect size is the difference between the medians (Non-Responders-Responders). Figure 4B also includes the 95% Confidence intervals. Between group comparisons were by Mann-Whitney U test. Abbreviations: MCID, minimal clinically important difference; LTBP2, latent-transforming growth factor beta-binding protein 2; TGFβ-1, transforming growth factor beta 1; FGF-2, basic fibroblast growth factor; TIMP-1, tissue inhibitor of metalloproteinases 1; TSG-6, tumour necrosis factor-inducible gene 6 protein; IL-6, interleukin 6; MCP-1, monocyte chemoattractant protein 1; IL-8, interleukin 8; MMP3, matrix metalloproteinase-3; KOOS, Knee Injury and Osteoarthritis Outcome Score (KOOS_4_ is composite measure of 4 domains).

|  | Activin A | LTBP2 | TGFβ-1 | FGF-2 | TIMP-1 | TSG-6 | | IL-6 | MCP-1 | IL-8 | MMP3 |
| --- | --- | --- | --- | --- | --- | --- | --- | --- | --- | --- | --- |
|  |  |  |  |  |  | |  |  |  |  |  |
| Activin A |  |  |  |  |  | |  |  |  |  |  |
| LTBP2 | 0.25 |  |  |  |  | |  |  |  |  |  |
| TGFβ-1 | -0.35* | 0.35* |  |  |  | |  |  |  |  |  |
| FGF-2 | <0.01 | -0.02 | 0.27 |  |  | |  |  |  |  |  |
| TIMP-1 | 0.07 | 0.15 | 0.07 | 0.05 |  | |  |  |  |  |  |
| TSG-6 | 0.41* | 0.20 | 0.01 | 0.01 | 0.35* | |  |  |  |  |  |
| IL-6 | 0.16 | 0.23 | 0.01 | -0.15 | 0.24 | | 0.79*** |  |  |  |  |
| MCP-1 | 0.35* | -0.45* | -0.45* | 0.35* | -0.06 | | 0.20 | 0.07 |  |  |  |
| IL-8 | 0.15 | 0.49* | -0.02 | -0.42* | -0.25 | | 0.47* | 0.55** | -0.16 |  |  |
| MMP3 | <0.01 | 0.45* | 0.04 | -0.03 | 0.69** | | -0.10 | 0.11 | -0.32* | -0.07 |  |

**Supplementary Figure 1. Correlation between change of analytes in the synovial fluid of participants over 3 weeks of knee joint distraction.**

Spearman rank tests were performed to determine correlations between the change in levels of synovial fluid analytes over 3 weeks of distraction period (concentration at 3 weeks-baseline concentrations). Correlation coefficients were calculated using all available participant data and the mean of 2 repeated (duplicate) measures for each synovial fluid sample.

Strength of correlation by Spearman R coefficient is shown:

* (Light grey shading): Low positive (negative) correlation, 0.30 to 0.49 (−0.30 to −0.49)

** (Mid grey shading): Moderate positive (negative) correlation, 0.50 to 0.69 (−0.50 to −0.69)

*** (Dark grey shading): High positive (negative) correlation, 0.70 to 0.89 (−0.70 to −0.89)

Abbreviations: LTBP2, latent-transforming growth factor beta-binding protein 2; TGFβ-1, transforming growth factor beta 1; FGF-2, basic fibroblast growth factor; TIMP-1, tissue inhibitor of metalloproteinases 1; TSG-6, tumour necrosis factor-inducible gene 6 protein; IL-6, interleukin 6; MCP-1, monocyte chemoattractant protein 1; IL-8, interleukin 8; MMP3, matrix metalloproteinase 3.

**Supplementary Figure 2.** Scatter plots showing the association between the change in synovial fluid analyte for each individual over the distraction period with the change in clinical outcome KOOS_4_ over 6 months, for each of the 10 analytes (regression statistics for these data are shown in middle panel of Figure 4A and Supplementary Table 2).

*

**Supplementary Figure 3**. The change of concentration in each analyte over the 6 week distraction period is shown (6 week level – baseline level), for 2 subgroups: Responders (those whose change in KOOS_4_ over 3 months was ≥ 10 points, i.e. those achieving the MCID for KOOS_4_); and Non-Responders (those whose change in KOOS_4_ over 3 months was <10 points, i.e. those not achieving the MCID for KOOS_4_). The bars represent the median and 95% Confidence Intervals for each group. Between group comparisons were by Mann-Whitney U test, *P=0.02. Abbreviations: MCID, minimal clinically important difference; LTBP2, latent-transforming growth factor beta-binding protein 2; TGFβ-1, transforming growth factor beta 1; FGF-2, basic fibroblast growth factor; TIMP-1, tissue inhibitor of metalloproteinases 1; TSG-6, tumour necrosis factor-inducible gene 6 protein; IL-6, interleukin 6; MCP-1, monocyte chemoattractant protein 1; IL-8, interleukin 8; MMP3, matrix metalloproteinase-3; KOOS, Knee Injury and Osteoarthritis Outcome Score (KOOS_4_ is composite measure of 4 domains).
