# Supplementary material for: The molecular profile of synovial fluid changes upon joint distraction and is associated with clinical response in knee osteoarthritis
Source: Osteoarthritis Cartilage. 2020 Mar;28(3):324–33. doi: 10.1016/j.joca.2019.12.005 (PMC7054834; doi:10.1016/j.joca.2019.12.005)
Supplement: Multimedia component 2 [file mmc2.pdf]

Analysis Plan

1. **Title:** Validation of putative biomarkers of successful cartilage regeneration in patients undergoing surgical joint distraction.

Version no: 1.6

Date submitted:

2. **Lead investigators:** Prof. Tonia Vincent; Dr. Fiona Watt; Dr. Andy Judge.

3. **Sponsoring senior investigator:** Prof. Tonia Vincent.

4. **Provisional list of potential co-authors:** Benjamin Hamid; Renata Hrusecka; Cesar Garriga, Simon Mastbergen; Tonia Vincent; Fiona Watt; Andy Judge; Floris P. J. G. Lafeber and TBD.

5. **Institutions:** NIHR Musculoskeletal Biomedical Research Unit, University of Oxford. Tissue Regeneration; Rheumatology & Clinical Immunology; University Medical Center Utrecht; The Netherlands. Arthritis Research UK Centre for Osteoarthritis Pathogenesis; Kennedy Institute of Rheumatology; NDORMS, University of Oxford.

6. **Research Questions:**

    1. Is there change in any markers, or the balance of markers in patients' synovial fluid pre and post joint distraction?

    2. If there is change, does this change have association with clinical outcome data (knee pain reduction, knee function, cartilage volume)?

7. **Hypothesis:**

    H_0: The selected biomarkers do not change between pre and post joint distraction.

    H_1: There is change in biomarkers (or some of them) between pre and post joint distraction.

H_0: Any change found in biomarkers is not clinically relevant.

H_1: There is clinically relevant change, of biomarkers (or some of them) between pre and post joint distraction, demonstrated by association with clinical outcome data.

8. Background:

There is some evidence than cartilage can ‘self-repair’ if any adverse mechanical environment of the joint is corrected[1]. There are molecules in the synovial fluid of patients which can be quantitated, and that could be potential markers of successful repair.

9. Sample:

Pilot study with 20 patients undergoing joint knee distraction in Utrecht, Netherlands.

10. Inclusion/Exclusion Criteria:

Inclusion criteria	Exclusion criteria
• Aged over 18 years.<br>• Consent competent and wiling to consent.	• <18 years.
• Patients who underwent joint knee distraction in Utrecht and provided baseline together with at least one follow up synovial fluid sample.<br>• Diagnosis of osteoarthritis.<br>• Approached October 2015-January 2017	

11. Main outcome variables:

A

- Biomarkers: activin A, latent-transforming growth factor beta-binding protein 2 (LTBP2), transforming growth factor beta 1 (TGFb1), basic fibroblast growth factor (FGF2), matrix metalloproteinase-3 (MMP-3), tumour necrosis factor-inducible gene 6 protein (TSG-6), tissue inhibitor of metalloproteinases 1 (TIMP-1), interleukin 6 (IL-6), monocyte chemoattractant protein 1 (MCP1 also known as CCL2), interleukin 8 (IL-8).
    - Difference between marker level at end of distraction period and at pre-distraction (baseline).
        - activina_6w_bl=activinaend- activinabl
        - ltbp2_6w_bl=ltbp2end- ltbp2bl
        - tgfb1_6w_bl=tgfb1end- tgfb1bl
        - fgf2_6w_bl=fgf2end- fgf2bl
        - timp1_6w_bl=timp1end- timp1bl
        - il6_6w_bl=il6end- il6bl
        - mcp1_6w_bl=mcp1end- mcp1bl
        - il8_6w_bl=il8end- il8bl
        - mmp3_6w_bl=mmp3_6w- mmp3bl
    - Relevant change between endpoint/midpoint and baseline concentrations was categorised into (0) no relevant change (1) relevant increment, (2) relevant decrement (do file “H thresholds KJD.do”)).

- Firstly, thresholds were obtained from the IQRs of concentration (mean of 2 measures per knee) of the biomarker[2]. Biomarker concentrations (baseline, 3 weeks on and 6 on) were classified into (0) normal [>=25 and <75 percentiles], (1) high [>=75 percentile] and (2) low [<25 percentile].

- Secondly, change between baseline concentration and endpoint (6 weeks following knee joint distraction) or baseline and midpoint (3 weeks) was evaluated as follows:

  i. (0) No relevant change: baseline = normal and endpoint/midpoint = normal; baseline = high and endpoint/midpoint = high; baseline = low and endpoint/midpoint = low.

  ii. (1) Relevant increment: baseline = normal and endpoint/midpoint = high; baseline = low and endpoint/midpoint = normal; baseline = low and endpoint/midpoint = normal.

  iii. (2) Relevant decrement: baseline = normal and endpoint/midpoint = low; baseline = high and endpoint/midpoint = normal; baseline = high and endpoint/midpoint = low.

B

- Pain, symptoms, physical activity and quality of life related to the injured knee:

  - Knee Injury and Osteoarthritis Outcome Score (KOOS), from which $KOOS_4$, a single composite score can be calculated (an average of 4 of the 5 KOOS subscales including Pain, Symptoms, Sports/Recreation and Quality of Life) after 1 year with knee distraction intervention. $KOOS_4$ will be divided in the best quartile (values≥ percentile 75) and the others (values <percentile 75). Change in $KOOS_4$ score:

$KOOS_4$ at 12 months – $KOOS_4$ at baseline. Hence positive values stand for improvement.

- Pain reduction:
    - Change in the domain of symptoms of “Knee injury and Osteoarthritis Outcome Score” (KOOS) after 1 year with knee distraction intervention.
    - Change in the domain of pain of “Knee injury and Osteoarthritis Outcome Score” (KOOS) after 1 year with knee distraction intervention.
- Better knee function:
    - Change in the domain of daily life of “Knee injury and Osteoarthritis Outcome Score” (KOOS) after 1 year with knee distraction intervention.
    - Change in the domain of sport of “Knee injury and Osteoarthritis Outcome Score” (KOOS) after 1 year with knee distraction intervention.
- Health status:
    - Change in EQ5D score after 1 year with knee distraction intervention.
    - Change in the domain of quality of life of “Knee injury and Osteoarthritis Outcome Score” (KOOS) after 1 year with knee distraction intervention.
- Cartilage volume: K/L grade change after 1 year with knee distraction intervention.

12. **Baseline Co-variables**, before knee joint distraction intervention *(field names are in italic)***:**

- Socio-demographic: *gender* (1=male, 2=female), *age* (years).

- Patient history: height (*height*: cm) weight (*weight*: kg); Body max index (*bmi*: kg/m^2). [Check outliers].

- Knee treatments and procedures: Kellgren and Lawrence grade (*kl*: 0 to 4 based on x-rays; kl2; 0=kl 0 to 3, 1=kl 4), affected knee (*knee*: 1=left, 2=right)

- Self-assessment:

  - Knee Injury and Osteoarthritis Outcome Score, KOOS (highest scores indicate no problems, ranges 0 to 100): symptom score (*koossympbl*: units); pain score (*koospainbl*: units); function in daily life score (*koosadlbl*: units); function in sport score (*koossportbl*: units); quality of life score (*koosqolbl*: units). (*koos4:* units, average score of the four subscale scores for *koos* Pain, *koos* Symptoms, *koos* Sport/Rec and *koos* QOL)[3].

  - WOMAC (highest scores indicate worse pain, stiffness, and functional limitations.): pain domain (*womacpainbl*: units, range 0 to 20); stiffness domain (*womacstifbl*: units, range 0 to 8); function score (*womacfuncbl*: units, range 0 to 68); sum of the 3 previous domains (*womactotalbl*: units, range 0 to 96)

  - Patient's state of health (EQ-5D) (*eq5dbl*, range 50 to 90 in our sample).

- Test: Synovial Fluid blood staining (*bloodbl*: 1=none, 2=mild, 3=moderate, 4=severe) or its binary version (*bloodbl2*: 0=none/mild, 1=moderate/severe)

- Biomarkers (grouped according to their putative role in joint) **mean of 2** repeated measures for each patient:

  - (1) Regenerative/ Anti-inflammatory/ Anti-catabolic:

- activin A (*activinabl*), "Activin A conc. (pg/ml)"; (*activinablng*), "Activin A conc. (ng/ml)"; (*activinablngz*), "Z value of Activin A conc. (ng/ml)"; (*activinablz*), "Z value of Activin A conc. (pg/ml)"; (*ltbp2blz*), "Z value of LTBP2 conc. (pg/ml)"; (activina*blcat*: 0= normal , 1= high, 2= low), “baseline concentrations"

- latent-transforming growth factor beta-binding protein 2 (*ltbp2bl*), "LTBP2 conc. (pg/ml)"; (*ltbp2blng*), "LTBP2 conc. (ng/ml)" ; (*ltbp2blngz*), "Z value of LTBP2 conc. (ng/ml)"; (*ltbp2blz*), "Z value of LTBP2 conc. (pg/ml)"; (*ltbp2blcat*: 0= normal [$\geq 25\%_{OMB}$ to $<75\%_{OMB}$], 1= high, 2= low), “baseline concentrations"

- transforming growth factor beta 1 (*tgfb1bl*), "TGFb1 conc. (pg/ml)"; (*tgfb1blng*), "TGFb1 conc. (ng/ml)"; (*tgfb1blngz*), “Z value of TGFb1 conc. (ng/ml)"; (*tgfb1blcat*: 0= normal, 1= high, 2= low), “baseline concentrations"

- basic fibroblast growth factor (*fgf2bl*), "FGF2 conc. (pg/ml)", there is also evidence of differential regulation for this protein; (*fgf2blz*), “Z value of FGF2 conc. (pg/ml)"; (*fgf2blcat*: 0= normal, 1= high, 2= low), “baseline concentrations"

- tissue inhibitor of metalloproteinases 1 (*timp1bl*), "TIMP-1 conc. (pg/ml)";_(*timp1blng*), "TIMP-1 conc. (ng/ml)";_(*timp1blngz*), “Z value of TIMP-1 conc. (ng/ml)"; (*timp1blcat*: 0= normal, 1= high, 2= low), “baseline concentrations"

- tumour necrosis factor-inducible gene 6 protein (*tsg6bl*), "TSG-6 conc. (pg/ml)";

- (2) Pro-inflammatory/Degradative:

  - interleukin 6 (*il6bl*), "IL-6 conc. (pg/ml)"; (*il6blz*), "Z value of IL-6 conc. (pg/ml)"; (*il6blcat*: 0= normal, 1= high, 2= low), "baseline concentrations"

  - monocyte chemoattractant protein 1 (*mcp1bl*), "MCP1 conc. (pg/ml)"; (*mcp1blz*), "Z value of MCP1 conc. (pg/ml)" ; (*mcp1blcat*: 0= normal, 1= high, 2= low), "baseline concentrations"

  - interleukin 8 (*il8bl*), "IL-8 conc. (pg/ml)"; (*il8blz*), "Z value of IL-8 conc. (pg/ml)"; (*il8blcat*: 0= normal, 1= high, 2= low), "baseline concentrations"

  - matrix metalloproteinase-3 (*mmp3bl*), "MMP-3 conc. (pg/ml)"; (*mmp3blng*), "MMP-3 conc. (ng/ml)"; (*mmp3blngz*), "Z value of MMP-3 conc. (ng/ml)"; (*mmp3blcat*: 0= normal, 1= high, 2= low), "baseline concentrations"

13. Proposed analytical approach:

Analysis 1 (not actually answering the study aims):

1.1. Description of variables, including putative biomarkers at baseline, before knee joint distraction. (do file "A descriptive JDK.do") (Excel file "Descriptive JDK 1.1.xlsx" spreadsheet "KJD")

1.1.1. Measures under the Lower Limit of Quantitation (LLOQ) will be discarded. E.g. Values under 62.5 pg/ml for TGFb1 will be treated as missing.

1.1.2. Measures above the Upper Limit of Quantitation (ULOQ) will be discarded. E.g. we will be treat as missing those values higher than 8000 pg/ml for TGFb1 or 5,000,000 pg/ml for MMP-3.

1.1.3. We will use the mean value of the two measures collected for each biomarker concentration at baseline. Therefore the unit of analysis will be the knee of the contributing patient. We will analyse the mean baseline value on each patient.

1.1.4. Percentage will be obtained for categorical variables in order to measure the frequency of each category. We will present the mean and its standard deviation (sd) for variables with normal distribution. For non-normal distributions we will utilise the median with the interquartile ranges (IQR) (**Table 1**). We will assessed normality with graphic representation (histograms and Q-Q plots). The Q-Q plots helps us to identify if the quantiles of our continuous variable are coincident with the quantiles of the normal distribution. We will complement the analysis of normality using the Shapiro–Wilk test[4]. However, if the graphics look roughly normal we will assume their result over the test.

Analysis 2: we will use non-parametric methods based on ranks because our dataset is small (n=20). Therefore, it is obvious non-normality cannot be corrected by a suitable transformation.

2. Association among markers at baseline, 3 weeks and 6 weeks.

2.1. We will see how correlations among biomarkers are different at baseline, midpoint (3 weeks) and endpoint (6 weeks). (do file “A descriptive JDK.do” lines 261, 294, 298) (Excel file “Descriptive JDK 1.1.xlsx” spreadsheet “Correlations”) (Table 3). We will expect to see different pairwise of biomarkers and also different size of correlation in the different time points (research question 1 - RQ 1). To do so, we will work out the Spearman's rank

correlation coefficients[5]. We will apply the following interpretation according to the size of the correlation[6]:

**Size of Correlation**	**Interpretation**
0.90 to 1.00 (−0.90 to −1.00)	Very high positive (negative) correlation
0.70 to 0.90 (−0.70 to −0.90)	High positive (negative) correlation
0.50 to 0.70 (−0.50 to −0.70)	Moderate positive (negative) correlation
0.30 to 0.50 (−0.30 to −0.50)	Low positive (negative) correlation
0.00 to 0.30 (0.00 to −0.30)	negligible correlation

2.2. We will check if there is association between the biomarker concentrations 6 weeks after the intervention and baseline variables including baseline concentrations of biomarkers (RQ 1). (do file “D correlation end baseline.do”) (Excel file “Descriptive JDK 1.1.xlsx” spreadsheet “Correlations”) (**Table 4**) To do so, we will work out the Spearman's rank correlation coefficients. We will exclude from this analysis measures of the same biomarker at different time points. To evaluate change on the same variable at different times in the same patients (non-independent samples) we will use the Wilcoxon signed rank test (see 2.4). If both variables have a continuous distribution we will calculate the Pearson product-moment correlation coefficient (Stata command pwcorr), i.e. the counterpart parametric test of the Spearman’s rank correlation coefficients. Note: Because EQ5D usually presents a bimodal distribution we will treat it as a non-normal variable.

2.3. Are some of the baseline variables related to the absolute value of change? (change= concentration at 6 weeks – baseline concentration) We will analyse the correlation between baseline variables, including baseline concentrations of biomarkers, and change in biomarker concentrations. (do file “E1 spearman.do” lines 7-25) (Excel file “correlation.xlsx” spreadsheet “Correlations”) To do so, we will run the Spearman's rank

correlation coefficients. We will exclude from this analysis comparisons on the same biomarker.

2.4. Is the absolute value of change related to the absolute value of change of other biomarkers? We will analyse the correlation in change vs. change, (change= concentration at 6 weeks – baseline concentration) (do file “E1 spearman.do” lines 48-51) (Excel file “correlation.xlsx” spreadsheet “Correlations”) To do so, we will run the Spearman's rank correlation coefficients. We will exclude from this analysis comparisons on the same biomarker.

2.5. We will check how much of the change is statistically significant at 6 weeks (endpoint) (RQ 1) (**Table 2**). To do so, we will assess the equality of matched pairs of observations (same biomarker collected from the same knee at different time points) using the Wilcoxon matched-pairs signed-ranks test[7]. The null hypothesis is that both distributions are the same. (do file “E1 spearman.do” lines 62-70, Stata command signrank) (Excel file “Descriptive JDK 1.1.xlsx” spreadsheet “Wilcoxon Rank at endpoint”). We will use the parametric counterpart paired t-test if the distribution of the biomarker is normal. Note: An experiment run on 40 different patients is not as powerful as a before-and-after comparison using the same 20 patients (see ranksum in Stata).

2.6. We will check how much of the change is statistically significant at 3 weeks (midpoint) (RQ 1) (**Table 2**). We like to see early changes in biomarker concentrations. To do so, we will assess the equality of matched pairs of observations (same biomarker collected from the same knee at different time points) using the Wilcoxon matched-pairs signed-ranks test[7]. The null hypothesis is that both distributions are the same. (do file “F signrank midpoint.do”) (Excel file “Descriptive JDK 1.1.xlsx” spreadsheet “Wilcoxon Rank at midpoint”). We will use the parametric counterpart paired t-test if the distribution of the biomarker is normal.

2.7. We will classify biomarkers in 4 groups with the information of the bullet points 2.3 and 2.4 (RQ 1): a) Change of concentration at 3 weeks and 6 weeks according to the baseline level, b) change of concentration at 3 weeks (early change) but not change at 6 weeks, c) change of concentration only at 6 weeks, d) no change over the time. (Excel file "Descriptive JDK 1.1.xlsx" spreadsheet "Wilcoxon Rank at endpoint") (Table 2 already shows this information).

2.8. Change in biomarkers between "no change", "increment" and "decrement" categories after 3 and 6 weeks and baseline (RQ 1). (do file "H thresholds KJD.do") (Excel file "Descriptive JDK 1.1.xlsx" spreadsheet "Change IQR categories") (**Figure 1, Figure 2**).

3. Analysis 3: Pattern recognition of synovial fluid biomarkers.According to two groups: (1) Regenerative/ Anti-inflammatory/ Anti-catabolic and (2) Pro-inflammatory/ Degradative. Association of markers at baseline regulated by FGF2 and CTGF, respectively.

3.2. Factor analysis (RQ 1). We will use this approach to find a few combinations of variables, called factors, that adequately explain the overall observed variation, and thus to reduce the complexity of the data. We will test the suitability of the data before the analysis using two test the Kaiser-Meyer-Olkin measure of sampling adequacy[8,9] and the Bartlett's test of Sphericity[10]. We will focus on the rotated factor matrix taken those values over 0.6 of each factor. Biomarkers with >0.6 in the same factor will be part of the same cluster. The factor plot in the rotated factor space will help us to see graphically the clusters. We will generate a table with the rotated factor matrix and a figure with the rotated factor space till 3 factors/dimensions.

3.2.1. The KMO test gives a measure that varies between 0 and 1, and values closer to 1 are better. A value of 0.6 is a suggested minimum. For reference, Kaiser put the following values on the results:

0.00 to 0.49	Unacceptable
0.50 to 0.59	Miserable
0.60 to 0.69	Mediocre
0.70 to 0.79	Middling
0.80 to 0.89	Meritorious
0.90 to 1.00	Marvellous

3.2.2. Bartlett's test of Sphericity tests the null hypothesis that the correlation matrix is an identity matrix. An identity matrix is matrix in which all of the diagonal elements are 1 and all off diagonal elements are 0. Small values (less than 0.05) of the significance level indicate that a factor analysis may be useful with our data.

4. Analysis 4: Logistic regression models of participants' $KOOS_4 \geq 75\%$ vs. $KOOS_4 < 75\%$ at 12 months. Simple linear regression between change over 12 months in $KOOS_4$ and baseline analytes. Spearman pairwise correlation of $KOOS_4$ at 12 months and change over 12 months in $KOOS_4$ according to a) baseline concentrations of analytes, b) change over 6 weeks in concentrations of analytes. (RQ 2). (do file "I KOOS4.do") (Excel file "Descriptive JDK 1.1.xlsx" spreadsheet "Table 5") (log file: "I KOOS4.log") (**Table 5**).Analysis 5 (suggested in email March 5 2018): Spearman pairwise correlation of change over 6 months and 3 months in $KOOS_4$ according to change over 6 weeks in concentrations of analytes. (RQ 2). (do file "I KOOS4.do") (Excel file "Descriptive JDK 1.2.xlsx" spreadsheet "Table 5") ("Table 6.pdf").Analysis 6 (suggested in email March 5 2018): Spearman pairwise correlation of change over 12 months in $KOOS_4$ according to baseline and 6 weeks in concentrations of analytes. (RQ 2). (do file "I KOOS4.do") (Excel file "Descriptive JDK 1.2.xlsx" spreadsheet "Table 7") ("Table 7.pdf").Analysis 8 (suggested in email March 5 2018): Simple linear regression between change over 12 months in $KOOS_4$ and categories of change (according to baseline interquartile distribution of concentrations) over 6 weeks in concentrations

of analytes. (RQ 2). (do file “I KOOS4.do”) (Excel file “Descriptive JDK 1.2.xlsx” spreadsheet “Figure 3”) (“p values fig 3.pdf”).

I will use Stata and SPSS.

---

14. If analysis involves using procedures/ measurements that are not included in original core protocol:

15. Limitations of the study

Due to the nature of the study (a pilot study) the number of participants is low.

16. Deadline for abstract/manuscript submission

17. Mock-up of key tables:

Table 1. Initial analytes considered from the grant application. FGF2/CTGF regulation information is included.

Molecule	Putative role in joint	Induced by uniaxial joint compression?	Induced by FGF2 in vitro?	Dependent upon CTGF in vitro?	Synovial fluid assay validated?
**Activin A**	Regenerative	Yes	Yes	No	Yes
**CCL2 (MCP-1)**	Unknown	No	No	No	Yes
**CTGF**	Regenerative	Unknown	Yes	Yes	No
**FGF2**	Regenerative	No	Unknown	No	Yes
**IL1b**	Degradative	No	No	No	Yes
**IL6**	Controversial	No	No	No	Yes
**LTBP2**	Regenerative	Unknown	Unknown	Yes	Yes
**MMP19**	Unknown	Yes	Yes	No	No
**MMP3**	Degradative	No	No	No	Yes
**Podoplanin**	Unknown	Yes	Yes	No	No
**TGFb1**	Regenerative	Unknown	Unknown	Yes	Yes
**TIMP1**	Anti-catabolic	Yes	Yes	No	Yes
**TSG6**	Anti-inflammatory	Yes	Yes	No	Yes

Table 2. Analytes used. FGF2/CTGF regulation information is included.

Molecule	Putative role in joint	Induced by uniaxial joint compression?	Induced by FGF2 in vitro?	Dependent upon CTGF in vitro?	Synovial fluid assay validated?
**Activin A**	Regenerative	Yes	Yes	No	Yes
**CCL2 (MCP-1)**	Unknown	No	No	No	Yes
**FGF2**	Regenerative	No	Unknown	No	Yes
**IL6**	Controversial	No	No	No	Yes
**IL8**	Controversial	Unknown	Unknown	Unknown	Yes
**LTBP2**	Regenerative	Unknown	Unknown	Yes	Yes
**MMP3**	Degradative	No	No	No	Yes
**TGFb1**	Regenerative	Unknown	Unknown	Yes	Yes
**TIMP1**	Anti-catabolic	Yes	Yes	No	Yes
**TSG6**	Anti-inflammatory	Yes	Yes	No	Yes

18. References

1 Wiegant, K. *et al.* Sustained clinical and structural benefit after joint distraction in the treatment of severe knee osteoarthritis. *Osteoarthritis and cartilage* **21**, 1660-1667, doi:10.1016/j.joca.2013.08.006 (2013).

2 Revicki, D., Hays, R. D., Cella, D. & Sloan, J. Recommended methods for determining responsiveness and minimally important differences for patient-reported outcomes. *Journal of clinical epidemiology* **61**, 102-109, doi:10.1016/j.jclinepi.2007.03.012 (2008).

3 Roos, E. M. & Toksvig-Larsen, S. Knee injury and Osteoarthritis Outcome Score (KOOS) - validation and comparison to the WOMAC in total knee replacement. *Health and quality of life outcomes* **1**, 17, doi:10.1186/1477-7525-1-17 (2003).

4 Royston, P. A toolkit for testing for non-normality in complete and censored samples. *The statistician*, 37-43 (1993).

5 Spearman, C. The proof and measurement of association between two things. *The American journal of psychology* **15**, 72-101 (1904).

6 Hinkle, D. E., Wiersma, W. & Jurs, S. G. *Applied Statistics for the Behavioral Sciences*. (Houghton Mifflin, 1994).

7 Wilcoxon, F. Individual comparisons by ranking methods. *Biometrics bulletin* **1**, 80-83 (1945).

8 Cerny, B. A. & Kaiser, H. F. A study of a measure of sampling adequacy for factor-analytic correlation matrices. *Multivariate behavioral research* **12**, 43-47 (1977).

9 Kaiser, H. F. An index of factorial simplicity. *Psychometrika* **39**, 31-36 (1974).

10 Harman, H. H. *Modern factor analysis*. (University of Chicago Press, 1976).
